# Efficacy, safety and circulating tumor cell dynamics in patients treated with Eribulin for HER2-negative advanced breast cancer – results from the phase II DETECT IVb trial

**DOI:** 10.1007/s10549-026-08069-2

**Published:** 2026-09-16

**Authors:** F. Schochter, T.W.P. Friedl, H. Schäffler, F. Meier-Stiegen, K.N. Pfister, S. Riethdorf, L. Wiesmüller, H. Neubauer, L. Müller, P. Wimberger, P.A. Fasching, R. Lorenz, T. Hempel, S. Heublein, A. Hartkopf, K. Pantel, B. Rack, V. Müller, T. Fehm, W. Janni, J.P. Cieslik

**Affiliations:** 1 Department of Obstetrics and Gynecology, Ulm University, 89075 Ulm, Germany; 2 Department of Obstetrics and Gynecology, University Hospital Düsseldorf, Düsseldorf, Germany; 3 Center for Integrated Oncology (CIO Aachen, Bonn, Cologne), Duesseldorf, Germany; 4 Institute of Tumor Biology, University Medical Center Hamburg-Eppendorf, 20246 Hamburg, Germany; 5 Studienzentrum UnterEms, Leer, Germany; 6 Department of Gynecology and Obstetrics, Technische Universität Dresden, Dresden, Germany; 7 7 NCT Dresden, Dresden, Germany; 8 Department of Gynecology and Obstetrics, Comprehensive Cancer Center Erlangen-EMN, University Hospital Erlangen, Friedrich-Alexander-Universität Erlangen-Nürnberg, Erlangen, Germany; 9 Studien GbR Braunschweig, Braunschweig, Germany; 10 Gesundheit Nord, Klinikumverbund Bremen, Bremen, Germany; 11 Department of Obstetrics and Gynecology, University of Tuebingen, Tuebingen, Germany; 12 Department of Gynecology and Obstetrics, University Hospital Hamburg- Eppendorf, Hamburg, Germany

**Keywords:** Metastatic breast cancer, Eribulin, Efficacy, Safety, Circulating tumor cells, CTC clearance

## Abstract

**Background:**

Eribulin is a non-taxane microtubule inhibitor that showed survival benefits for pretreated metastatic breast cancer (MBC) patients. Circulating tumor cells (CTCs) are a prognostic marker, but their role in predicting response to specific MBC treatments is less clear. In this trial, we assessed the clinical outcome and its association with CTC-dynamics in HER2-negative MBC treated with eribulin.

**Patients and methods:**

HER2-negative MBC-patients were screened for CTCs within the DETECT study program using the CellSearch® technology (Menarini Silicon Biosystems; Bologna, Italy). Patients with HER2-negative CTCs were eligible for the single-arm DETECT-IVb study treatment with eribulin. Primary endpoint was progression-free survival (PFS), and secondary endpoints included overall survival (OS), CTC-clearance rate and safety.

**Results:**

Median PFS and OS were 4.6 months and 13.4 months, respectively. Multivariable adjusted analyses showed that patients with CTC-clearance at first follow-up (34.7%) had significantly improved PFS (p = 0.043) but not OS (p = 0.192) compared to patients with at least 1 CTC. Likewise, patients with CTC-clearance at the end of the treatment (23.1%) achieved a significantly better PFS (p = 0.015) but not OS (p = 0.218). Neutropenia, leukopenia, anemia and fatigue were the most common adverse events and no new or unexpected safety signals were obtained.

**Conclusion:**

In this trial, we could confirm eribulin as an effective treatment option in high-risk HER2-negative MBC. CTCs proved their role as a prognostic marker and our results support the potential role of CTC dynamics as an early biomarker of treatment response to eribulin.

**Trial registration:**

EudraCTNo2013-001269-18 http://ClinicalTrials.gov, TRN NCT02035813, Registration date 2014-01-12.

**Supplementary Information:**

The online version contains supplementary material available at https://doi.org/10.1007/s10549-026-08069-2.

## Introduction

Therapies in incurable metastatic breast cancer (MBC) focus on prolonging survival and maintaining quality of life. According to current guidelines, single-agent chemotherapy is the preferred treatment option if chemotherapy is indicated [1]. Eribulin is a non-taxane, microtubule dynamic inhibitor with both antimitotic activities and non-mitotic effects on tumor biology, tumor environment, and epithelial–mesenchymal transition (EMT) [2–5], which significantly improved overall survival (OS) in pretreated MBC patients [6–8]. Given the role of eribulin in the therapy of MBC, it is important to further investigate the molecular mechanisms and to identify biomarkers predictive of eribulin response.

Circulating tumor cells (CTCs) showed their prognostic value in several MBC studies [9]. Although there is some evidence that repeated CTC measurements can predict therapy response [10–12], until now no clinical trial using CTC-enumeration as a decision tool for therapeutic interventions has succeeded. The DETECT study program uses an alternative approach, where not only the number but also phenotypic characteristics of CTCs are considered for the choice of individualized treatment options [13, 14]. 

The DETECT IVb study reported here focuses on MBC patients with HER2-negative disease according to both primary tumor histology and CTCs and evaluates treatment efficacy of eribulin in these patients. A particular interest was to analyze outcome in relation to CTC prevalence, dynamics and clearance rates under therapy to assess the utility of CTCs as an early treatment monitoring tool for MBC patients receiving eribulin.

## Materials and methods

### DETECT study program

The phase II DETECT IV trial (EudraCT Number 2013-001269-18, ClinicalTrials.gov Identifier NCT02035813) is part of the prospective, multicenter DETECT study program for patients with MBC [14]. The DETECT study program involves an initial CTC-screening to determine presence and HER2-status of CTCs in patients with HER2-negative MBC, who could not be treated by surgery or radiotherapy alone. CTC-positive patients (at least one CTC in 7.5 m of blood) with only HER2-negative CTCs could be recruited in the DETECT IV trial, which comprises two cohorts (DETECT IVa and DETECT IVb) that represent two separate and independent single-arm studies. In DETECT IVb, which is reported here, patients with either triple-negative tumors or hormone receptor positive tumors and indication for chemotherapy received single-agent chemotherapy with eribulin (HALAVEN^®^). The DETECT IV trial was performed in accordance to the Declaration of Helsinki, adhered to good clinical practice and was approved by the responsible sponsor’s ethics committee (MC-LKP-668).

### CTC enumeration and assessment of HER2 status on CTCs

CTC assessments in the DETECT study program were performed using the CellSearch technology (Menarini Silicon Biosystems, Bologna, Italy) as described in Riethdorf et al. [15]. Peripheral blood samples of 7.5 ml were collected into CellSave Tubes and sent to CTC laboratories, where they were analyzed within 96 h. HER2 expression was categorized based on an immunohistochemistry (IHC) score as negative (IHC 0), weak (IHC 1+), moderate (IHC 2+) or strong (IHC 3+) according to Riethdorf et al. [16]. To be eligible for inclusion in the DETECT IVb study, patients had to be CTC-positive with all CTCs showing HER2-negativity (defined as IHC score 0, 1 + or 2+).

### Patient recruitment, study treatment and assessment phase

Patients were recruited from March 2015 to October 2019 and provided written informed consent. Eribulin doses are administered according to the European prescribing information 1.23 mg/m^2^ with possible dose reductions to 0.97 or 0.62 mg/m^2^; these are equivalent to 1.4, 1.1, and 0.7 mg/m² eribulin mesylate as used in the US prescribing information. Treatment duration was up to 12 months, with a 24-month follow-up. Study visits were scheduled every six weeks, radiological tumor evaluation per RECIST 1.1 every 12 weeks. Blood sampling for CTC-analysis was recommended at tumor evaluations and at the end of the treatment period.

Safety was evaluated by the frequency of adverse events (AEs) and serious adverse events (SAEs) graded according to international Common Terminology Criteria for Adverse Events (CTCAE), version 4.0.

### Statistical analysis

The primary endpoint was progression-free survival (PFS); secondary end points included overall survival (OS), CTC clearance rate, and safety. CTC clearance rate was defined as proportion of patients with no evidence of detectable CTCs (0 CTCs/7.5 mL blood) during follow-up among all patients (CTC positivity at baseline was one of the inclusion criteria; see above). CTC counts were further categorized according to the established prognostic cutoff described by Cristofanilli et al. (< 5 vs. ≥5 CTCs/7.5 mL blood), defining low and high CTC burden, respectively [17]. CTCs were assessed at two follow-up timepoints: first on-treatment follow-up (≥ 21 days after registration) and at the end of study treatment. Baseline characteristics were summarized using absolute and relative frequencies for categorical and ordinal variables, and using median and range for continuous variables. Associations between CTC status and categorical clinical parameters were assessed using chi square tests; in case of expected frequencies of less than 5 in 2 × 2 contingency tables, Fisher’s exact test was used instead. PFS and OS were calculated from registration to progressive disease or death, respectively, with patients without known events being censored at the date of last adequate follow-up. Survival outcomes were estimated using the Kaplan–Meier method and compared by log-rank tests. Univariable and multivariable Cox proportional hazards regression models were used to estimate hazard ratios (HRs) with 95% confidence intervals (CIs) for predefined prognostic factors including metastatic treatment line (first vs. second vs. third or higher), metastasis-free interval (≤ 12 months, including de novo metastatic disease vs. > 12 months), and site of first metastasis (bone, no visceral vs. visceral, no bone vs. bone and visceral vs. other). To test whether the addition of the variable CTC status (at baseline and/or during follow up) significantly improved the model, we added CTC status to the multivariable Cox regression model and used the likelihood-ratio test to assess changes in model fit. There was no significant time-by-variable interaction for any of the included variables (all *p* > 0.10), indicating that the proportional hazard assumption was not violated. Statistical analyses were performed using SPSS version 26 (IBM Corp, Armonk, New York, USA). All tests were two-sided with a significance level α of *p* < 0.05, and no adjustments of the significance level for multiple testing were made.

## Results

### CTC screening for participation in the DETECT IVb study

Overall, 1091 patients with HER2-negative MBC were screened for CTCs within the DETECT study program starting in December 2014. Due to low recruitment rate, enrollment for DETECT IVb was terminated prematurely in October 2019. Rather than the planned 120 patients, the final intention-to-treat (ITT) population for DETECT IVb comprised 109 registered patients. (CONSORT flow diagram, Supplementary Fig. 1).

### Patient baseline and tumor characteristics

The median age of enrolled patients was 62 years, and 87.2% of patients were postmenopausal. Most patients (75.2%) had hormone receptor-positive (luminal-like) tumors, while 20.2% had triple-negative disease. A metastasis-free interval of more than 12 months was observed in 84.4% of patients. Eribulin was administered as first-line treatment for MBC in 28.4% of patients. Detailed baseline characteristics are summarized in Table 1.

**Table 1:** Baseline patient and tumor characteristics

Variable	Total (*n* = 109)
Age at registration (median, range)	62 (34–81)
Menopausal status	
* premenopausal*	10 (9.2%)
* postmenopausal*	95 (87.2%)
* sterilization*	4 (3.7%)
Eastern Cooperative Oncology Group (ECOG) Performance Status	
* 0*	64 (58.7%)
* 1*	38 (34.9%)
* 2*	7 (6.4%)
Tumor subtype^1^	
* triple-negative (hormone receptor negative)*	22 (20.2%)
* luminal-like (hormone receptor positive)*	82 (75.2%)
* missing* ^*2*^	5 (4.6%)
Grading	
* G I*	2 (1.8%)
* G II*	64 (58.7%)
* G III*	35 (32.1%)
* missing* ^*2*^	8 (7.3%)
Histology	
* ductal*	70 (64.2%)
* lobular*	11 (10.1%)
* other*	28 (25.7%)
Site of first metastatic disease	
* bone (no visceral)*	43 (39.4%)
* visceral (no bone)*	28 (25.7%)
* bone and visceral*	14 (12.8%)
* other (no bone*,* no visceral)*	24 (22.0%)
Metastasis-free interval (from primary diagnosis to first metastatic disease)	
* ≤ 12 months* ^*3*^	17 (15.6%)
* > 12 months*	92 (84.4%)
Therapeutic setting (number of chemotherapy lines for metastatic disease)	
* 0*	31 (28.4%)
* 1*	33 (30.3%)
* ≥ 2*	45 (41.3%)

^1^ all patients with HER2 negative metastatic breast cancer

^2^ indicates that the respective information was unavailable in the source data and could not be determined retrospectively

^3^ including 15 cases with metastatic disease (pM1) at primary diagnosis (de novo metastatic disease)

### Termination of study treatment, progression-free survival and overall survival

Study treatment with eribulin was discontinued in 98 patients (89.9%). The most frequent reason for treatment discontinuation was progress (71 patients), followed by non-haematological toxicity (7 patients), patient withdrawal (6 patients), treatment delay (5 patients), death (4 patients), haematological toxicity (2 patients) and other reasons (3 patients).

**Fig. 1:**
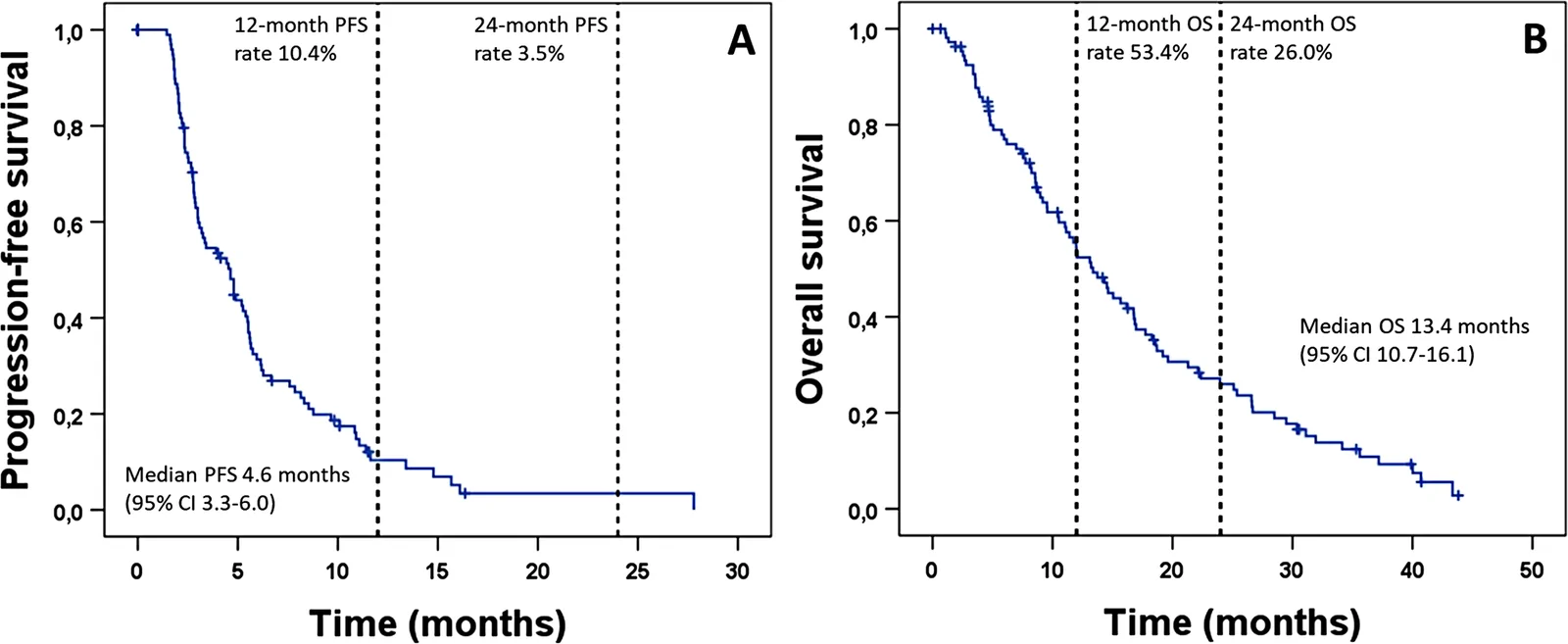
Kaplan-Meier survival plots for progression-free survival (**A**) and overall survival (**B**)

Disease progression occurred in 87 patients (79.8%). Median PFS was 4.6 months (95% CI 3.3–6.0 months), with 1-year and 2-year PFS rates of 10.4% and 3.5%, respectively (Fig. 1A). Overall, 88 patients (80.7%) died. Median OS was 13.4 months (95% CI 10.7–16.1 months); the 1-year and 2-year OS rates were 53.4% and 26.0%, respectively (Fig. 1B). One patient with disease progression was censored for OS at the date of last contact because the exact date of death was unknown.

Log-rank tests, univariable and adjusted multivariable Cox proportional hazard models showed that only metastatic treatment line but not metastasis-free interval, site of first metastatic disease, patient age and tumor subtype significantly affected PFS and OS in our patient cohort (Supplementary Results 1, Supplementary Tables 1, 2).

### Safety

In total, 1343 adverse events (AEs) of any grade and 181 AEs of grade ≥ 3 were observed during the study. Two patients did not receive study treatment because of withdrawal (one case) or death (one case) before the first study visit. Overall, 105 (96.3%) patients had at least one AE of any grade and 73 patients (67.0%) had at least one grade 3–5 AE. Adverse events occurring in ≥ 10% of patients (any grade) and AEs grade ≥ 3 occurring in ≥ 2% of patients are presented in Table 2.

**Table 2:** Most common adverse events. Shown are adverse events (total number of AEs observed, number and percentage of patients affected) of all grades affecting at least 10% of patients and adverse events grade 3 or higher affecting at least 2% of patients

NCI chapter title	Adverse event term	All grades			Grades 3/4/5		
		*n*	*n* patients affected	% patients affected	*n*	*n* patients affected	% patients affected
Blood and lymphatic system disorders	Anemia	89	27	24,8%	10	6	5,5%
Gastrointestinal disorders	Constipation	39	31	28,4%	1	1	0,9%
Gastrointestinal disorders	Diarrhea	14	12	11,0%	1	1	0,9%
Gastrointestinal disorders	Mucositis oral	23	18	16,5%	0	0	0,0%
Gastrointestinal disorders	Nausea	49	33	30,3%	0	0	0,0%
Gastrointestinal disorders	Vomiting	13	12	11,0%	2	2	1,8%
General disorders and administration site conditions	Fatigue	82	59	54,1%	7	6	5,5%
General disorders and administration site conditions	Flu like symptoms	13	12	11,0%	0	0	0,0%
General disorders and administration site conditions	Pain	14	11	10,1%	3	3	2,8%
Hepatobiliary disorders	Portal vein thrombosis	5	5	4,6%	4	4	3,7%
Investigations	Alanine aminotransferase increased	12	10	9,2%	4	4	3,7%
Investigations	Aspartate aminotransferase increased	15	11	10,1%	3	3	2,8%
Investigations	Neutrophil count decreased	112	32	29,4%	47	23	21,1%
Investigations	White blood cell decreased	55	25	22,9%	24	17	15,6%
Metabolism and nutrition disorders	Anorexia	24	17	15,6%	1	1	0,9%
Musculoskeletal and connective tissue disorders	Back pain	13	11	10,1%	1	1	0,9%
Musculoskeletal and connective tissue disorders	Bone pain	25	20	18,3%	1	1	0,9%
Musculoskeletal and connective tissue disorders	Pain in extremity	19	13	11,9%	1	1	0,9%
Nervous system disorders	Headache	22	16	14,7%	0	0	0,0%
Nervous system disorders	Peripheral sensory neuropathy	49	38	34,9%	4	4	3,7%
Psychiatric disorders	Depression	13	11	10,1%	0	0	0,0%
Psychiatric disorders	Insomnia	20	17	15,6%	1	1	0,9%
Respiratory, thoracic and mediastinal disorders	Cough	13	11	10,1%	0	0	0,0%
Respiratory, thoracic and mediastinal disorders	Dyspnea	40	26	23,9%	5	4	3,7%
Respiratory, thoracic and mediastinal disorders	Pneumonitis	5	5	4,6%	3	3	2,8%
Skin and subcutaneous tissue disorders	Alopecia	55	51	46,8%	0	0	0,0%
Vascular disorders	Hot flashes	21	17	15,6%	0	0	0,0%
Vascular disorders	Thromboembolic event	11	11	10,1%	3	3	2,8%

The most frequent AEs of any grade observed in more than 25% of patients were fatigue (54.1%), alopecia (46.8%), peripheral sensory neuropathy (34.9%), nausea (30.3%), neutropenia (29.4%), constipation (28.4%), and anemia (24.8%). The most common grade ≥ 3 or higher observed in more than 10% of patients were neutropenia (21.1%) and leukopenia (15.6%).

Overall, 71 serious adverse events (SAE) were reported in 49 patients. Most frequently reported SAEs were dyspnea (5 patients), pleural effusion (5 patients), febrile neutropenia (3 patients) and febrile leukopenia (1 patient). A possible relationship to eribulin treatment was reported for 25 SAEs, and no suspected unexpected serious adverse reaction (SUSAR) occurred.

There were 89 deaths, predominantly due to disease progression (81 cases). In six cases, the cause of death was unknown. Two deaths (stroke and subdural hemorrhage) were assessed as not related to study treatment.

### CTC status at baseline

At baseline, the median CTC count was 5 CTCs (range 1–482), consistent with the inclusion criterion for this study; 59 patients had ≥ 5 CTCs. Baseline CTC status (< 5 CTCs/≥ 5 CTCs) did not differ significantly according to tumor subtype (*p* = 0.170), metastatic treatment line (*p* = 0.222), metastasis-free interval (*p* = 0.915), or site of first metastatic disease (*p* = 0.966).

Baseline CTC status (≥ 5 CTCs vs. 1–4 CTCs) was not significantly associated with PFS in univariable analysis (HR 1.31, 95% CI 0.85–2.01; Supplementary Fig. 2A) but significantly associated with OS (HR 1.72, 95% CI 1.12–2.65; Supplementary Fig. 2B). Inclusion of baseline CTC status in the multivariable Cox regression model - adjusted for metastatic treatment line, metastasis-free interval, site of first metastatic disease, tumor subtype and patient age - significantly improved model fit for both PFS (*p* = 0.041) and OS (*p* = 0.001). Baseline CTC status (≥ 5 CTCs vs. 1–4 CTCs) remained independently associated with outcome, with hazard ratios of 1.63 (95% CI 1.02–2.62) for PFS and 2.25 (95% CI 1.36–3.74) for OS (Tables 3 and 4).

**Table 3:** Results of a multivariable Cox regression model for progression-free survival, including CTC status at baseline

Variable	Hazard ratio	95% CI	*P*-value
Age (years)	1.02	0.99–1.04	0.124
Tumor subtype			
* Luminal-like vs. triple negative*	0.50	0.27–0.92	0.026
Metastatic treatment line			0.015
* Second vs. first*	2.03	1.09–3.77	0.025
* Third or higher vs. first*	2.53	1.33–478	0.004
Metastasis-free interval			
* > 12 months vs. ≤ 12 months (including de novo metastatic disease)*	0.96	0.44–2.08	0.907
Site of first metastatic disease			0.153
* visceral (no bone) vs. bone (no visceral)*	1.07	0.60–1.92	0.811
* bone and visceral vs. bone (no visceral)*	0.68	0.29–1.64	0.391
* other vs. bone (no visceral)*	1.79	0.98–3.25	0.056
CTC status at baseline			
* ≥ 5 CTCs vs. 1–4 CTCs* ^*1*^	1.63	1.02–2.62	0.043

^1^ Please note that CTC-positivity (at least one CTC/7.5 mL blood) at baseline was an inclusion criterion for the DETECT IVb study

**Table 4:** Results of a multivariable Cox regression model for overall survival, including CTC status at baseline

Variable	Hazard ratio	95% CI	*P*-value
Age (years)	1.02	0.99–1.04	0.167
Tumor subtype			
* Luminal-like vs. triple negative*	0.56	0.30–1.05	0.070
Metastatic treatment line			0.120
* Second vs. first*	1.77	0.94–3.31	0.075
* Third or higher vs. first*	1.80	0.98–3.31	0.057
Metastasis-free interval			
* > 12 months vs. ≤ 12 months (including de novo metastatic disease)*	1.27	0.63–2.54	0.508
Site of first metastatic disease			0.135
* visceral (no bone) vs. bone (no visceral)*	2.04	1.11–3.74	0.021
* bone and visceral vs. bone (no visceral)*	1.43	0.66–3.01	0.364
* other vs. bone (no visceral)*	1.67	0.87–3.20	0.126
CTC status at baseline			
* ≥ 5 CTCs vs. 1–4 CTCs* ^*1*^	2.25	1.36–3.74	0.002

^1^ Please note that CTC-positivity (at least one CTC/7.5 mL blood) at baseline was an inclusion criterion for the DETECT IVb study

### CTC status at first on-treatment follow-up

At least one follow-up CTC assessment ≥ 21 days after registration was available for 75 patients (68.8%), with a median interval of 33 days (interquartile range 27–66 days, range 21–268 days). Two patients withdraw from the study before day 21, 3 died and 12 had progressive disease before a first on-treatment CTC assessment (≥ 21 days post-registration), while 17 had no first on-treatment CTC assessment for other reasons. Among the 75 patients with available follow-up data 53 (70.7%) had their first on-treatment CTC assessment between day 21 and 63 (approximately chemotherapy cycles 2 and 3), and 68 (90.7%) of these patients had their first on-treatment CTC assessment between day 21 and 84 (approximately chemotherapy cycles 2 to 4). The time of first on-treatment CTC assessment and end-of-treatment CTC assessment, progressive disease and end of study (death / censored) for each patient is shown in a swimmer plot (Supplementary Fig. 3). Median CTC-count at first on-treatment follow-up was 1 CTC (range 0–331). CTC clearance was observed in 26 patients (34.7%) and 49 patients (65.3%) remained CTC positive (at least one CTC), including 21 patients ≥ 5 CTCs. Compared with baseline, CTC count decreased in 54 patients (72.0%; median decrease 5 CTCs; range 1–450 CTCs), increased in 13 patients (17.3%; median increase 13 CTCs (range 3–311 CTCs) and remained unchanged in 8 (10.7%) patients. CTC clearance (yes/no) at first on-treatment follow-up was not significantly associated with tumor subtype (*p* = 0.864), metastatic treatment line (*p* = 0.379), metastasis-free interval (*p* = 0.523), or site of first metastatic disease (*p* = 0.720). Similarly, no significant associations were found between CTC status at first on-treatment follow-up using the </≥ 5 CTCs cutoff and tumor subtype (*p* = 1.000), metastatic treatment line (*p* = 0.440), metastasis-free interval (*p* = 1.000), or site of first metastatic disease (*p* = 0.935).

**Fig. 2:**
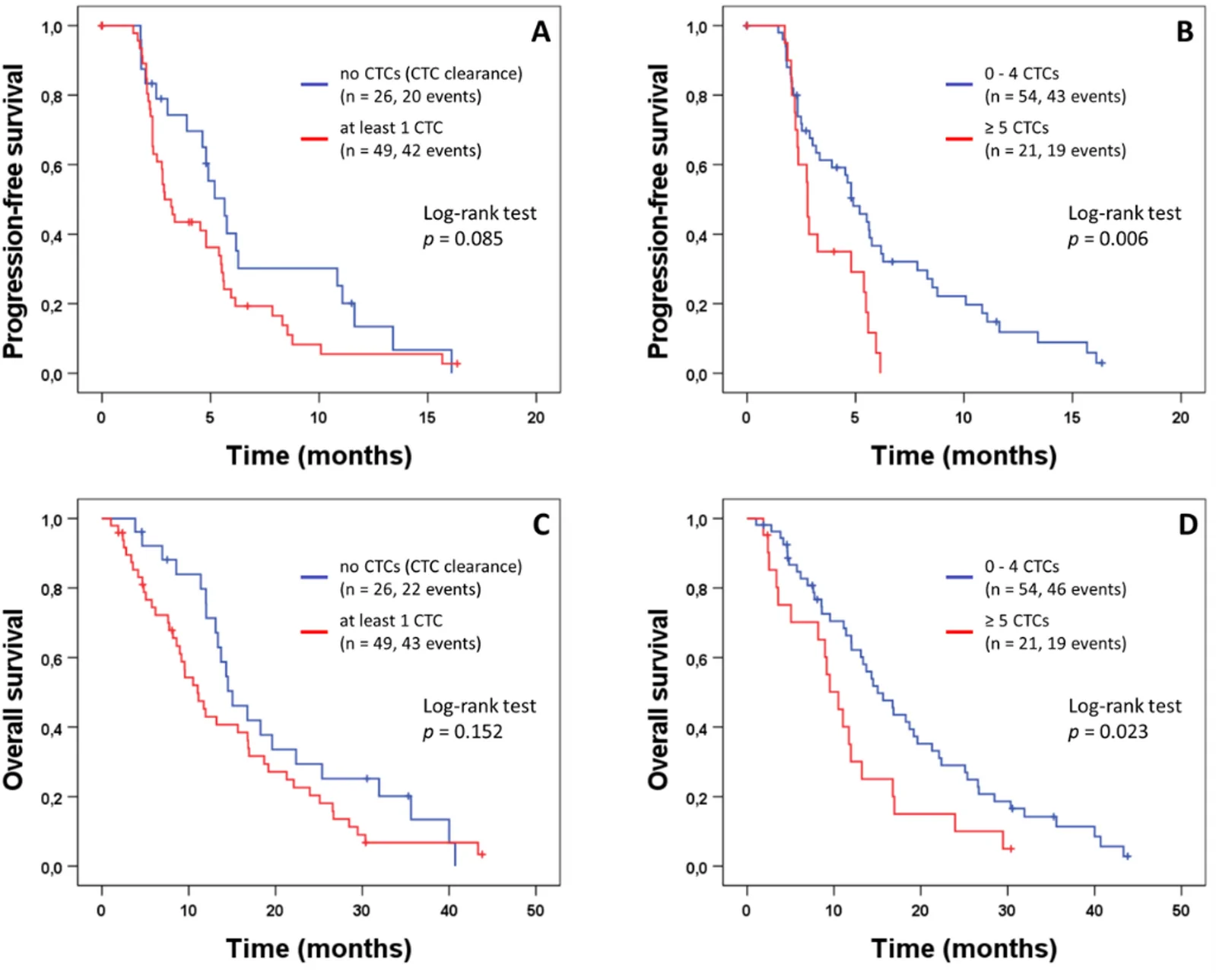
Kaplan-Meier plots for progression-free survival (**A** and **B**) and overall survival (**C** and **D**) according to CTC status at the time of first follow-up CTC assessment (*n* = 75)

Patients with CTC clearance at the first on-treatment follow-up CTC assessment showed numerically longer PFS (median 5.7 vs. 2.9 months; HR 0.63; 95% CI 0.36–1.08; Fig. 2A) and OS (median 15.0 vs. 11.0 months; HR 0.69; 95% CI 0.41–1.15; Fig. 2C) compared with patients with detectable CTCs. Using the predefined cut-off of ≥ 5 CTCs, patients with 0–4 CTCs had significantly improved PFS (median 4.9 vs. 2.8 months; HR 0.45; 95% CI 0.25–0.81; Fig. 2B) and OS (median 15.0 vs. 10.5 months; HR 0.53; 95% CI 0.31–0.92; Fig. 2D) compared to patients with ≥ 5 CTCs.

In adjusted multivariable analyses, complete CTC clearance was significantly associated with improved PFS (HR 0.56; 95% CI 0.31–0.98; *p* = 0.043). Patients with 0–4 CTCs at the first on-treatment follow-up showed a borderline significant association with improved PFS (HR 0.55; 95% CI 0.30–1.01; *p* = 0.052). A similar pattern was observed for OS. Complete CTC clearance was associated with a non-significant reduction in risk of death (HR 0.69; 95% CI 0.39–1.21; *p* = 0.192), whereas patients with 0–4 CTCs at the first on-treatment follow-up had a significantly improved OS compared to those with ≥ 5 CTCs (HR 0.45; 95% CI 0.25–0.82; *p* = 0.009).

To evaluate the potential impact of variable timing of the first on-treatment CTC assessment being linked to confounding factors related to outcome, we repeated the multivariable analyses including only the 68 patients with a first assessment between 21 and 84 days post-registration, thereby excluding delayed assessment. Results were consistent with the primary analyses. Complete CTC clearance was significantly associated with improved PFS (HR 0.44; 95% CI 0.23–0.85; *p* = 0.014), while a CTC count < 5 CTCs at the first on-treatment follow-up was almost significantly associated with improved PFS (HR 0.55; 95% CI 0.29–1.04; *p* = 0.065). Regarding OS, complete CTC clearance was associated with a non-significant reduction in mortality risk (HR 0.66; 95% CI 0.36–1.23; *p* = 0.192), whereas a CTC count < 5 CTCs at the first on-treatment follow-up remained significantly associated with improved OS compared to those with ≥ 5 CTCs (HR 0.38; 95% CI 0.20–0.73; *p* = 0.003).

### CTC status at end of study treatment

Only 39 patients had a CTC assessment at the end of study treatment. Median CTC count at the end of treatment was 5 CTCs (range 0–875). CTC clearance was detected in 9 patients (23.1%), and 20 patients (51.3%) had ≥ 5 CTCs. CTC clearance at the end of study treatment was significantly associated with improved PFS (median 13.4 vs. 5.4 months, HR 0.26, 95% CI 0.07–0.89) but not OS (median 15.7 vs. 12.0 months, HR 0.49, 95% CI 0.20–1.20). More details regarding CTC status at the end of study treatment and survival are presented in Supplementary Results 2.

## Discussion

The phase II single-arm DETECT IVb trial evaluated efficacy and safety of eribulin monotherapy in a high-risk cohort of patients with HER2-negative MBC and baseline CTC-positivity, including patients with triple-negative or hormone receptor-positive disease with indication for chemotherapy. Another aim was to assess the prognostic value and treatment-associated dynamics of CTCs at baseline, during treatment and at the end of study treatment.

### Efficacy

Median PFS and OS reported in DETECT IVb were 4.6 months and 13.4 months, respectively, and were consistent with data on eribulin in MBC. In the 305/EMBRACE trial where eribulin was used as 3rd line treatment, median PFS and OS were 3.7 months and 13.1 months, respectively [6]. Real-world data from a Belgian expanded access program reported comparable outcomes with a median PFS of 3.2 months and OS of 11.3 months in patients pretreated with taxane, anthracycline and capecitabine [18].

The DETECT IVb population is even better comparable to the patients enrolled in 301-trial, which included first- and second-line treatment in the advanced setting [7]. The 301-population included 19% first-line, 54% second-line and 27% further-line patients, and the reported median PFS and OS were 4.1 and 15.9 months. The slightly longer OS observed in the 301 study may be explained by a higher proportion of later-line patients and the inclusion criterion of CTC positivity at baseline, reflecting a particular high-risk profile for our DETECT IVb population. Observational data of early-line use (49% first line, 30% second line, 21% third line) from Japan showed even better PFS (4.9 months) and OS (17.4 months) compared to the 301 study [19], supporting the impact of treatment-line and baseline risk on clinical outcome.

### Safety

The safety profile observed in DETECT IVb was consistent with reported randomized trials and real-world studies, summarized in a recent meta-analysis [20]. The most clinically relevant adverse events were hematologic toxicities and peripheral neuropathy. Rates of grade ≥ 3 neutropenia (21%) were comparable to the EMBRACE-study (21%) and the 301-study (25%). Four patients (4%) presented with febrile leukopenia, which is also in the range observed in the EMBRACE-trial (5%) and the 301-study (2%).

Peripheral neuropathy of any grade was observed in 35% of patients, which is similar to the EMBRACE (34.6%) but slightly higher than the 301-study (27.4%). Data from the prospective IRENE-study suggest that a substantial proportion of neuropathy events observed during treatment may represent worsening of pre-existing neuropathy related to prior taxane-exposure rather than de novo eribulin-induced peripheral neuropathy [21]. This highlights the importance of detailed baseline documentation when interpreting neuropathy rates across studies.

Other common adverse events, including anemia, fatigue, nausea and alopecia occurred at frequencies comparable to previous reports [6, 7, 20], and no new safety signals were identified. Treatment discontinuation due to toxicity occurred in 8.3% patients, consistent to previous trials including the 301-study (7.9%) and the EMBRACE-study (13%).

### Prognostic value and treatment-associated dynamics of CTCs

The prognostic relevance of CTCs at the start of a new treatment line in MBC is well established [9], and baseline CTC number can be used to stratify MBC into an aggressive or indolent disease using a cutoff of 5 CTCs [17]. Applying the threshold of ≥ 5 CTCs to our patient population, baseline CTC-burden was independently associated with both PFS and OS in multivariable analysis, confirming its prognostic relevance.

One of the endpoints analyzed in our study was the response of CTCs to chemotherapy in terms of CTC clearance and their potential role as an early biomarker of treatment response. We could show that CTC clearance was significantly associated with increased PFS, both when assessed early in the treatment phase and when assessed at the end of study treatment. Several other studies also revealed an association between repeated CTC enumeration results during treatment and outcome in MBC [22, 23]. These findings support the concept of CTC-dynamics as an on-treatment biomarker and are in line with previous studies demonstrating CTC counts obtained during treatment may be more informative than baseline enumeration alone.

A large pooled analysis including 4436 patients further demonstrated that CTC responders (converting from CTC-positive at baseline to CTC-negative at first follow-up) had considerably improved OS compared to CTC-non-responders, independent of tumor subtypes and treatment modality [12]. Collectively, these results indicate that CTC-dynamics during treatment provides important information and is a robust prognostic marker in MBC.

Despite this evidence, the clinical utility of repeated CTC monitoring to guide treatment decisions remains controversial in MBC. While the French STIC CTC trial suggested that CTC-guided treatment decisions may be beneficial in hormone receptor-positive, HER2-negative MBC [24], both the SWOG-S0500 and the CirCe01 trial failed to demonstrate a survival benefit from early treatment change based on persistent CTC-positivity [25, 26]. These results underline that CTC-enumeration alone may be insufficient to guide therapeutic decisions [27] and highlight the need for deeper biological characterization of functional or molecular CTC features. In smaller analyses, Smerage et al. highlighted the role of apoptosis markers [28], and Schochter et al. the role of 53BP1 signals [29] on detected CTCs. Together with our findings, this supports a model in which CTC dynamics serve as a prognostic and response biomarker, while future clinical utility may depend on integrating quantitative and qualitative CTC analyses.

### Eribulin, CTCs and epithelial-to-mesenchymal transition - Outlook and future research

To further elucidate effects of eribulin on CTCs and outcome, additional research is needed to better understand both its antimitotic and non-antimitotic mechanism of action. Beyond microtubule inhibition, eribulin has been shown to modulate epithelial-to-mesenchymal transition (EMT), a dynamic and reversible epigenetic process characterized by transcriptional and phenotypic changes that confer migratory, invasive and therapy-resistant properties to tumor cells [30, 31]. EMT plays a role in dissemination and is reversed through mesenchymal-to-epithelial transition (MET) [32]. Preclinical studies have confirmed that eribulin induces MET-like changes in triple-negative breast cancer cells, accompanied by alterations in gene expression and signaling pathways associated with reduced invasiveness [5, 33].

Furthermore, accumulating evidence indicates a complex interplay between EMT and DNA damage repair pathways [34]. First promising results were obtained with CTC expression of the DNA repair protein 53BP1, highlighting the potential role of CTC-specific 53BP1 signals as a tool to predict responsiveness in eribulin-treated MBC patients [29].

Future studies integrating longitudinal CTC profiling with molecular and functional characterization may help to clarify the mechanisms underlying eribulin activity and to identify biomarkers that might even be a tool to predict eribulin responsiveness [35].

### Limitations of the study

Several limitations of this study have to be considered. First and foremost, the findings of our study have to be interpreted as exploratory and hypothesis-generating rather than confirmatory. Second, the single-arm study design precludes definitive conclusions regarding treatment efficacy or causality, and comparison relies on data from other clinical trials. Third, prior taxane exposure was not included as a covariate because detailed information on individual prior taxane treatment was unavailable, precluding robust multivariable adjustments. Although eribulin exhibits limited cross-resistance with taxanes [2], residual confounding cannot be excluded, even if previous studies suggest no major impact of prior taxane therapy on eribulin efficacy [6, 7].

In addition, there are also limitations with respect to CTC analyses. CTC-assessments at first on-treatment follow-up and at the end of treatment were not available for all patients, often due to treatment discontinuation. This resulted in a decreasing number of evaluable samples and limited the robustness of longitudinal analyses. Importantly, missing CTC data may not be missing at random, as patients that died early, had early progression or declining performance status were unable or less likely to undergo follow-up CTC assessments. Such a bias – broadly referred to as immortal-time bias or guarantee-time bias - has also been reported previously and might impact conclusions based on results regarding the clinical relevance of numerical and phenotypic changes in CTCs [36, 37]. The interpretation of longitudinal CTC analyses is also limited by the variable timing of follow-up CTC assessments and the lack of correlation with radiological response. Finally, only CTC enumeration was assessed longitudinally, whereas there was no data available regarding serial characterization of CTC phenotypes (e.g., HER2 status or EMT-associated markers). Such data may provide additional clinically relevant information, and these aspects should be addressed in future prospective studies.

## Conclusion

The results of the DETECT IVb trial demonstrate clinical benefit of eribulin in a high-risk cohort of patients and assure the role of eribulin as a safe and important option in pretreated MBC patients. Furthermore, CTC clearance at the first on-treatment follow-up was associated with significantly improved PFS, suggesting that CTC dynamics may reflect early treatment response. Prospective, biomarker-driven interventional trials are warranted to determine whether early CTC changes can be used to guide treatment decisions in eribulin-treated MBC.

## Supplementary Information

Below is the link to the electronic supplementary material.

Supplementary Material 1

## Data Availability

The datasets generated and analysed during the current study are not publicly available due to data privacy reasons but are available from the corresponding author on reasonable request.

## Declarations

### Generative AI and AI-assisted technologies in the manuscript preparation process

During the preparation of this manuscript, the authors used ChatGPT for language refinement and phrasing. After using this tool/service, the authors reviewed and edited the content as needed and take full responsibility for the content of the published article.
