# Supplementary material for: Efficacy, safety and circulating tumor cell dynamics in patients treated with Eribulin for HER2-negative advanced breast cancer – results from the phase II DETECT IVb trial
Source: Breast Cancer Res Treat. 2026 Sep 16;219(3):12. doi: 10.1007/s10549-026-08069-2 (PMC13582096; doi:10.1007/s10549-026-08069-2)
Supplement: Supplementary file 1 [file 10549_2026_8069_MOESM1_ESM.docx]

**SUPPLEMENT**

**Supplementary Results 1.** Baseline patient and tumor characteristics and survival

**Supplementary Results 2.** CTC status at end of study treatment and survival

**Supplementary Table 1.** Results of a multivariable Cox regression model for progression-free survival

**Supplementary Table 2.** Results of a multivariable Cox regression model for overall survival

**Supplementary Figure 1*.*** CONSORT flow chart of the DETECT study-program.

**Supplementary Figure 2*.*** Kaplan-Meier plots for progression-free survival (A) and overall survival (B) according to CTC status at baseline (n = 109).

**Supplementary Figure 3*.*** Per-patient swimmer plot illustrating time of first on-treatment CTC assessment, end of study treatment CTC assessment, progressive disease and end of study.

**Supplementary Figure 4*.*** Kaplan-Meier plots for progression-free survival (A) and overall survival (B) according to CTC status at end of study treatment (n = 39).

**Supplementary Results 1.** Baseline patient and tumor characteristics and survival

Using both log-rank tests. and univariable and adjusted multivariable Cox proportional hazard models, we tested whether known prognostic factors for MBC patients were also prognostic in our high-risk DETECT IVb patient cohort. Metastatic treatment line (first vs second vs third or higher) significantly affected both PFS and OS (log-rank test: p = 0.013 and p = 0.025, respectively). Pairwise comparisons showed that first metastatic treatment line patients had better PFS and OS than second and third or higher metastatic treatment line patients (first vs. second: p = 0.012 and p = 0.048, respectively; first vs. third or higher: p = 0.009 and p = 0.012, respectively), while no significant differences in PFS or OS were observed between second line and third or higher treatment line patients (p = 0.955 and p = 0.965, respectively). Hazard ratios for second line vs first line patients were 2.16 (95% CI 1.20 – 3.89) for PFS and 1.96 (95% CI 1.08 – 3.56) for OS, and hazard ratios for third or higher line vs first line patients were 2.16 (95% CI 1.22 – 3.81) for PFS and 2.03 (95% CI 1.18 – 3.49) for OS. In contrast to the metastatic treatment line, we found no significant effect of the metastasis-free interval (≤ 12 months vs. > 12 months) on PFS or OS (p = 0.199 and p = 0.262). Likewise, no significant effect of site of first metastatic disease (bone, no visceral vs. visceral, no bone vs. bone and visceral vs. other) on PFS or OS was detected (p = 0.118 and p = 0.265). We also ran multivariable Cox regression models to evaluate which factors exhibit an independent effect on survival when other factors were accounted for. In addition to the variables metastatic treatment line, metastasis-free interval and site of first metastatic disease we also included patient age and tumor subtype. The results of the multivariable analyses, which are presented in Supplementary Table S1 for PFS and Supplementary Table S2 for OS, showed that only the metastatic treatment line significantly affected PFS and OS in our patient cohort.

**Supplementary Results 2.** CTC status at end of study treatment and survival

A CTC assessment at the end of study treatment was performed in 39 patients, and in 36 (92.3%) of these patients, study treatment was terminated prematurely (29 cases due to progress, 6 cases due to toxicity and one case due to other reasons). Median number of CTCs at the end of study treatment was 5 CTCs (range 0 – 875). No CTCs (that is, CTC clearance at the end of study treatment) were detected in 9 (23.1%) patients, and 20 (51.3%) patients had 5 or more CTCs. The three patients with no premature end of study treatment showed no CTCs in two cases and 29 CTCs in one case.

CTC clearance at the end of study treatment was associated with significantly improved PFS (median PFS 13.4 vs 5.4 months, HR 0.26, 95% CI 0.07 – 0.89; Supplementary Figure 3A) and numerically but not significantly improved OS (median OS 15.7 vs 12.0 months, HR 0.49, 95% CI 0.20 – 1.20; Supplementary Figure 3C) compared to patients with at least one CTC. With the 5 CTC cutoff, the pattern was reversed in the way that patients with 0 – 4 CTCs at the end of study treatment only had a numerically but not significantly improved PFS compared to patients with 5 or more CTCs (median PFS 5.7 vs. 3.2 months, HR 0.59, 95% CI 0.29 – 1.23; Supplementary Figure 3B), while the OS benefit became significant (median OS 16.8 vs 11.1 months, HR 0.47, 95% CI 0.23 – 0.93; Supplementary Figure 3D). The results of the adjusted multivariable Cox regressions closely resembled the results of the univariable analyses. Patients with CTC clearance at the end of study treatment showed significantly better PFS (HR 0.12; 95% CI 0.02 – 0.66; p = 0.015) but not OS (HR 0.46; 95% CI 0.13 – 1.59; p = 0.218) compared to patients with at least one CTC. Patients with 0 – 4 CTCs at the end of study treatment showed significantly better OS (HR 0.27; 95% CI 0.09 – 0.80; p = 0.017) but not PFS (HR 0.65; 95% CI 0.24 – 1.77; p = 0.396) compared to patients with at least 5 CTCs.

**Supplementary Table 1.** Results of a multivariable Cox regression model for progression-free survival

| **Variable** | **Hazard ratio** | **95% CI** | **P-value** |
| --- | --- | --- | --- |
| Age (years) | 1.02 | 0.99 – 1.04 | 0.186 |
| Tumor subtype  *Luminal-like vs triple negative* | 0.57 | 0.31 – 1.03 | 0.063 |
| Metastatic treatment line  *Second vs first*  *Third or higher vs first* | 2.02  2.79 | 1.09 – 3.76  1.48 – 5.26 | 0.007  0.026  0.002 |
| Metastasis-free interval  *> 12 months vs ≤ 12 months (including de novo metastatic disease)* | 0.96 | 0.44 – 2.05 | 0.905 |
| Site of first metastatic disease  *visceral (no bone) vs bone (no visceral)*  *bone and visceral vs bone (no visceral)*  *other vs bone (no visceral)* | 1.09  0.74  1.86 | 0.61 – 1.96  0.32 – 1.75  1.04 – 3.34 | 0.132  0.769  0.498  0.038 |

**Supplementary Table 2.** Results of a multivariable Cox regression model for overall survival

| **Variable** | **Hazard ratio** | **95% CI** | **P- value** |
| --- | --- | --- | --- |
| Age (years) | 1.01 | 0.99 – 1.04 | 0.229 |
| Tumor subtype  *Luminal-like vs triple negative* | 0.71 | 0.39 – 1.30 | 0.268 |
| Metastatic treatment line  *Second vs first*  *Third or higher vs first* | 1.76  2.10 | 0.94 – 3.29  1.16 – 3.82 | 0.048  0.078  0.015 |
| Metastasis-free interval  *> 12 months vs ≤ 12 months (including de novo metastatic disease)* | 1.31 | 0.66 – 2.62 | 0.443 |
| Site of first metastatic disease  *visceral (no bone) vs bone (no visceral)*  *bone and visceral vs bone (no visceral)*  *other vs bone (no visceral)* | 1.78  1.51  1.41 | 0.99 – 3.22  0.69 – 3.27  0.75 – 2.63 | 0.262  0.055  0.301  0.286 |

**Supplementary Figure 1*.*** CONSORT flow chart of the DETECT study-program.

**Supplementary Figure 2*.*** Kaplan-Meier plots for progression-free survival (A) and overall survival (B) according to CTC status at baseline (n = 109).

**Supplementary Figure 3*.*** Per-patient swimmer plot illustrating time and result (CTC clearance yes/no) of first on-treatment CTC assessment and end of study treatment CTC assessment, progressive disease and end of study.

**Supplementary Figure 4.** Kaplan-Meier plots for progression-free survival (A and B) and overall survival (C and D) according to CTC status at the end of study treatment (n = 39).
